# Clear Cell Carcinoma Arising From Adenomyotic Cyst: A Case Report

**DOI:** 10.7759/cureus.71503

**Published:** 2024-10-14

**Authors:** Yoshito Morishita, Hiroshi Yagi, Ichiro Onoyama, Hideaki Yahata, Kiyoko Kato

**Affiliations:** 1 Department of Obstetrics and Gynecology, Graduate School of Medical Sciences, Kyushu University, Fukuoka, JPN

**Keywords:** adenomyotic cyst, clear cell cancer, endometrial cancer (ec), lymph node metastasis, uterine adenomyosis

## Abstract

Clear cell carcinoma often arises from endometriosis, primarily from ovarian chocolate cysts and much less frequently from adenomyosis. We herein report a case of clear cell carcinoma arising from adenomyotic cyst in a 38-year-old woman, gravida 0, para 0, who was referred to our department with a diagnosis of a uterine tumor. Her medical history was unremarkable. Contrast-enhanced magnetic resonance imaging revealed a 13-cm uterine tumor with a predominantly hypointense signal on both T1- and T2-weighted images, accompanied by a hyperintense lesion in the center on T1-weighted images. Additionally, typical uterine leiomyomas were observed. Her serum CA125 (reference range: 0-35 units/ml) and CA19-9 (reference range: 0-37 units/ml) levels were elevated to 1,200 and 8,178 U/mL, respectively. Degenerated myoma was suspected preoperatively. Given the patient’s desire to preserve her fertility, a myomectomy was performed. Macroscopically, the tumor was solid, white, and fibroid-like but contained chocolate-colored fluid. Pathological examination revealed clear cell carcinoma characterized by adenocarcinoma cells with clear and eosinophilic cytoplasm and nuclear atypia arranged in a tubulocystic pattern. Endometriosis was also found within the tumor. Subsequently, a hysterectomy, bilateral salpingo-oophorectomy, and pelvic and para-aortic lymphadenectomy were performed. No malignancy was detected in the resected uterus or adnexa, but metastasis to a para-aortic lymph node was observed. Based on these findings, the patient was diagnosed with stage IIIC2 endometrial cancer (pT1bN2M0, clear cell carcinoma) and received postoperative adjuvant therapy with paclitaxel and carboplatin. Eighteen months later, her serum CA125 increased to 45 U/mL, and a contrast-enhanced computed tomography scan revealed multiple liver and left supraclavicular lymph node metastases. Five cycles of pembrolizumab and lenvatinib followed by four cycles of doxorubicin and cisplatin were ineffective. The patient died 13 months after the diagnosis of recurrence.

## Introduction

Clear cell carcinoma is a highly malignant histopathological subtype that exhibits resistance to standard chemotherapy [1]. Clear cell carcinoma often arises from endometriosis, a condition where tissues similar to the inner lining of the uterus grow outside the uterine cavity, frequently forming ovarian cysts known as “chocolate cysts”. Adenomyosis, on the other hand, is a pathological condition characterized by the presence of endometrial glands and stroma within the uterine myometrium accompanied by adjacent smooth muscle hyperplasia [2]. In most cases, adenomyotic lesions are diffusely scattered throughout the myometrium and rarely form cystic lesions. These cystic lesions, referred to as adenomyotic cysts, cystic adenomyoma, or cystic adenomyosis, are uncommon [3-8]. While clear cell carcinoma commonly arises from endometriosis, its occurrence in adenomyotic cysts is exceedingly rare. We herein present a case of clear cell carcinoma originating from a uterine adenomyotic cyst, highlighting its clinical presentation, diagnostic challenges and aggressive nature, thereby adding to the limited knowledge of clear cell carcinoma associated with adenomyotic cysts.

## Case presentation

**Figure 1:**
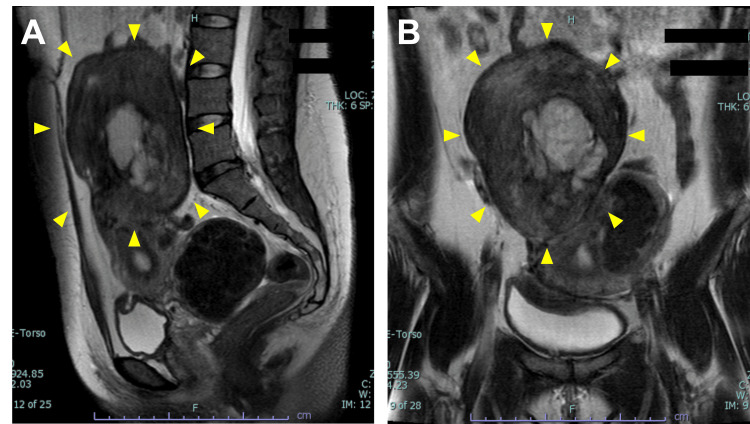
Preoperative magnetic resonance imaging of the pelvis Sagittal (A) and coronal (B) section of magnetic resonance imaging of the pelvis. T2-weighted images showed a 13-cm subserosal tumor (yellow arrowheads) with a hypointense signal. A hyperintense T2 signal was observed in the center of the tumor, suggesting a hemorrhagic component.

The patient was a 38-year-old woman, gravida 0, para 0, with an unremarkable medical and family history. She initially presented to an obstetrics and gynecology clinic with lower abdominal pain. Pelvic magnetic resonance imaging (MRI) revealed a 12-cm solid uterine tumor. Her serum CA125 (reference range: 0-35 units/ml) and CA19-9 (reference range: 0-37 units/ml) levels were elevated at 1,200 and 8,178 U/mL, respectively. She was referred to our department for further evaluation and treatment. Contrast-enhanced MRI confirmed a 13-cm subserosal tumor (Figure 1).

The tumor was predominantly solid but contained heterogeneous areas. It appeared hypointense on both T1- and T2-weighted images, with hyperintensity in the center suggesting a hemorrhagic component. No solid portion was found within the cystic area. Typical leiomyomas were also observed. Because the tumor had a smooth, well-defined margin with no evidence of infiltration into surrounding structures, it was initially diagnosed as a degenerated leiomyoma. Although the CA125 and CA19-9 levels were high, they were trending downward by the time the patient visited our department. The serum lactate dehydrogenase level was within the reference range. Based on these findings, our preoperative diagnosis was degenerated leiomyoma with multiple leiomyomas.

**Figure 2:**
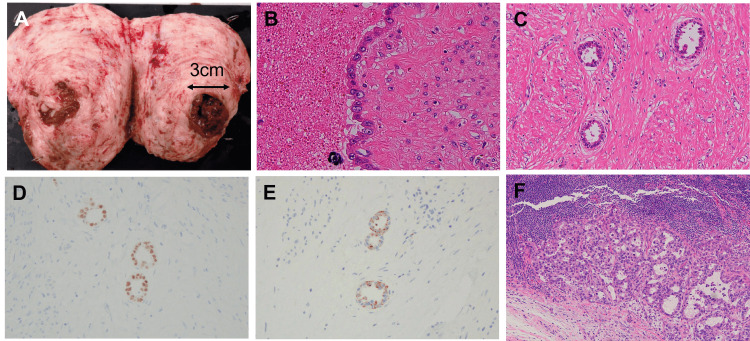
Macroscopic and pathological findings (A) Macroscopically, the uterine tumor was solid, white, and fibroid-like, but it contained a chocolate-like fluid inside. (B, C) Pathologically, the tumor showed proliferation of adenocarcinoma cells with clear and eosinophilic cytoplasm and nuclear atypia. (D, E) Immunohistochemically, the adenocarcinoma cells were positive for HNF-1b (D) and Napsin A (E). (F) Para-aortic lymph nodes were metastasized by carcinoma cells.

Given the patient’s desire to preserve her fertility, myomectomy was performed. Macroscopically, the tumor was solid, white, and fibroid-like, but it contained chocolate-colored fluid (Figure 2A). Pathological examination revealed proliferation of adenocarcinoma cells with clear and eosinophilic cytoplasm and nuclear atypia (Figure 2B). These cells were arranged in a tubulocystic pattern (Figure 2C). Immunohistochemical staining showed that the carcinoma cells were positive for HNF-1β and Napsin A (Figure 2D, 2E), focally positive for p53, and negative for ER and PgR. These findings confirmed a diagnosis of clear cell carcinoma. Endometriosis and hemosiderin-laden macrophages were also observed. Based on these results, the postoperative diagnosis was clear cell carcinoma arising from an adenomyotic cyst.

Staging laparotomy was subsequently performed. Intraoperative ascitic cytology was negative, and there was no evidence of peritoneal dissemination. Macroscopically, swelling of the pelvic and para-aortic lymph nodes was observed. Pathological analysis showed no residual carcinoma cells in the uterus, bilateral adnexa, omentum, or appendix. No metastases were found in the pelvic lymph nodes; however, carcinoma cells were present in 2 of 11 para-aortic lymph nodes (Figure 2F). Although no clear cancerous lesion was found in the endometrium, the final diagnosis was stage IIIC2 endometrial cancer. Postoperative adjuvant therapy with paclitaxel and carboplatin was initiated.

**Figure 3:**
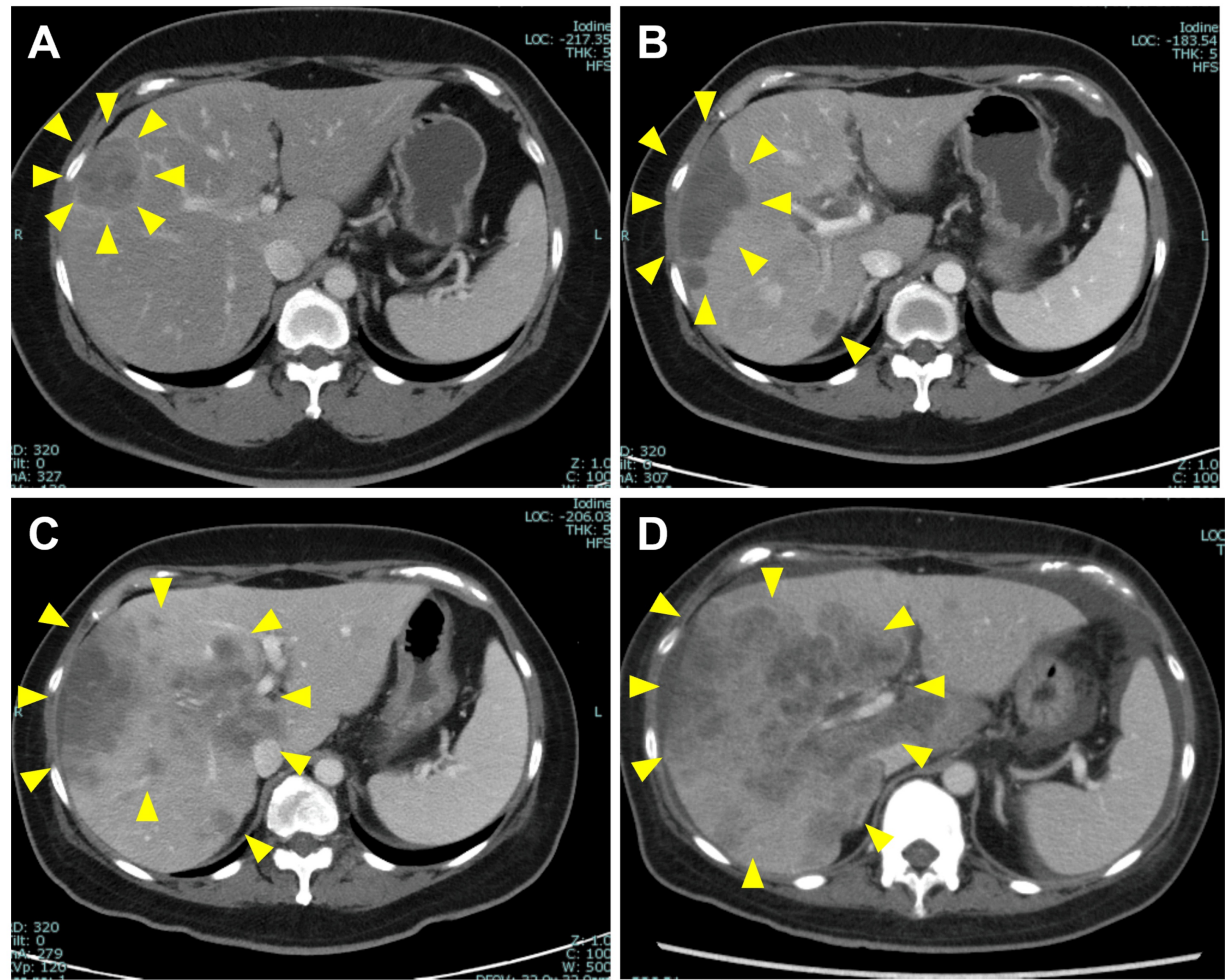
Computed tomography images of liver metastases (A) Twenty months after the primary treatment, computed tomography revealed multiple liver metastases. Computed tomography scans of the liver metastases were taken after (B) five cycles of lenvatinib plus pembrolizumab, (C) four cycles of doxorubicin and cisplatin, and (D) two cycles of paclitaxel and carboplatin. Yellow arrowheads indicate liver metastases.

Twenty-two months after completing chemotherapy, a contrast-enhanced computed tomography scan revealed multiple liver metastases as well as metastasis to the left supraclavicular lymph node (Figure 3A). At this point, the serum CA125 and CA19-9 levels were 45 U/mL and 18.9 U/mL, respectively. A diagnosis of recurrent stage IIIC2 endometrial cancer was made. Because the tumor exhibited microsatellite stability, the patient was treated with pembrolizumab and lenvatinib. After five cycles of this regimen, a follow-up contrast-enhanced computed tomography scan showed progression of the liver metastases (Figure 3B). Subsequently, the patient received four cycles of doxorubicin and cisplatin followed by two cycles of paclitaxel and carboplatin; however, none of these regimens proved effective (Figure 3C, 3D). The patient died 13 months after the diagnosis of recurrence.

## Discussion

Adenomyosis is a common gynecologic disorder, characterized by the presence of ectopic endometrial glands and stroma located deep within the myometrium, leading to hyperplasia of the adjacent smooth muscle [2]. Although rare, ectopic endometrial tissue within adenomyotic foci can serve as a precursor to cancer. In 1959, Colman and Rosenthal reported the following three diagnostic criteria for endometrial cancer arising in adenomyosis (EC-AIA): the absence of carcinoma in the endometrium or elsewhere in the pelvis, demonstration of carcinoma arising from the epithelium of adenomyosis and not from invasion by other sites, and the presence of endometrial stromal cells surrounding the epithelial glands, supporting the diagnosis of adenomyosis [9].

Because of its rarity, the clinicopathological characteristics of EC-AIA remain poorly understood. A systematic review of 37 reported cases of EC-AIA showed that 23 (85%) cases occurred in postmenopausal women, with abnormal genital bleeding being the most common clinical presentation [10]. Most cases were preoperatively misdiagnosed as uterine sarcoma, atypical leiomyoma, leiomyoma, or degenerated leiomyoma. Histologically, 20 (57.1%) cases were classified as endometrioid carcinoma, 5 (14.3%) as clear cell carcinoma, 4 (11.4%) as serous carcinoma, 4 (11.4%) as adenosarcoma, 1 (2.8%) as carcinosarcoma, and 1 (2.8%) as Müllerian mucinous borderline tumor. In two cases, the histological subtype was not reported. According to the FIGO 2008 staging system, 26 cases were stage I-II and 11 cases were stage III-IV. Among the advanced-stage cases, five had pelvic and/or para-aortic lymph node metastases and six had metastases to other sites. Immunohistochemical analysis revealed abnormal p53 expression in 10 of 20 cases examined. Recurrence was observed in 8 of the 23 cases with available follow-up.

In most cases, adenomyotic lesions diffusely spread throughout the myometrium. Adenomyosis with cystic lesions larger than 1 cm is rare and has been described as adenomyotic cyst, cystic adenomyoma, or cystic adenomyosis [3-8]. While EC-AIA is rare, cases of cancer originating from adenomyotic cysts, as in the present case, are even less frequently reported [11, 12].

Six cases of clear cell carcinoma arising from adenomyosis have been reported to date [12-15]. The chief complaints varied and included genital bleeding, abdominal pain, weight loss, and fever. Preoperative endometrial brush cytology was negative in all cases. Six of seven cases, including the present case, were initially diagnosed as degenerated leiomyoma. Given the rarity of this condition, specific ultrasound or MRI features have not been identified, making preoperative diagnosis challenging. In our case, although the marked elevation of CA125 and CA19-9 levels raised a concern for malignancy, subsequent decrease in these serum tumor markers preoperatively suggested a possible benign nature, which contributed to the initial misdiagnosis. However, in retrospect, these markers can fluctuate in malignancies arising from endometriosis, indicating their potential but limited diagnostic utility in differentiating between benign and malignant uterine tumors. The prognosis is difficult to predict because of the limited number of cases; however, distal metastases were observed in three of the seven cases, including ours.

## Conclusions

Although rare, ectopic endometrium within adenomyotic foci can serve as a precursor to cancer. Uterine cancer arising from adenomyotic cysts, an uncommon manifestation of adenomyosis, is even less frequently reported. We have herein presented a case of clear cell carcinoma originating from an adenomyotic cyst that demonstrated a poor prognosis. These clinical features - lower abdominal pain, a large solid uterine mass with unique MRI characteristics, significantly elevated serum tumor markers - could guide clinicians in considering adenomyotic cysts with malignant potential in similar cases.
